# The Characteristics and Motivations of Online Health Information Seekers: Cross-Sectional Survey and Qualitative Interview Study

**DOI:** 10.2196/jmir.1600

**Published:** 2011-02-23

**Authors:** John Powell, Nadia Inglis, Jennifer Ronnie, Shirley Large

**Affiliations:** ^2^NHS DirectBerrywood Business VillageSouthamptonUnited Kingdom; ^1^Warwick Medical SchoolUniversity of WarwickCoventryUnited Kingdom

**Keywords:** information seeking behaviour, information seeking behaviour, internet, patient-provider communication, health services research

## Abstract

**Background:**

Most households in the United Kingdom have Internet access, and health-related Internet use is increasing. The National Health Service (NHS) Direct website is the major UK provider of online health information.

**Objective:**

Our objective was to identify the characteristics and motivations of online health information seekers accessing the NHS Direct website, and to examine the benefits and challenges of the health Internet.

**Methods:**

We undertook an online questionnaire survey, offered to users of the NHS Direct website. A subsample of survey respondents participated in in-depth, semistructured, qualitative interviews by telephone or instant messaging/email. Questionnaire results were analyzed using chi-square statistics. Thematic coding with constant comparison was used for interview transcript analysis.

**Results:**

In total 792 respondents completed some or all of the survey: 71.2% (534/750 with data available) were aged under 45 years, 67.4% (511/758) were female, and 37.7% (286/759) had university-level qualifications. They sought information for themselves (545/781, 69.8%), someone else (172/781, 22.0%), or both (64/781, 8.2%). Women were more likely than men to seek help for someone else or both themselves and someone else (168/509 vs 61/242, χ^2^_2_ = 6.35, *P* = .04). Prior consultation with a health professional was reported by 44.9% (346/770), although this was less common in younger age groups (<36 years) (χ^2^_1_ = 24.22, *P* < .001). Participants aged 16 to 75 years (n = 26, 20 female, 6 male) were recruited for interview by telephone (n = 23) and instant messaging/email (n = 3). Four major interview themes were identified: motivations for seeking help online; benefits of seeking help in this way and some of the challenges faced; strategies employed in navigating online health information provision and determining what information to use and to trust; and specific comments regarding the NHS Direct website service. Within the motivation category, four concepts emerged: the desire for reassurance; the desire for a second opinion to challenge other information; the desire for greater understanding to supplement other information; and perceived external barriers to accessing information through traditional sources. The benefits clustered around three theme areas: convenience, coverage, and anonymity. Various challenges were discussed but no prominent theme emerged. Navigating online health information and determining what to trust was regarded as a “common sense” activity, and brand recognition was important. Specific comments about NHS Direct included the perception that the online service was integrated with traditional service provision.

**Conclusions:**

This study supports a model of evolutionary rather than revolutionary change in online health information use. Given increasing resource constraints, the health care community needs to seek ways of promoting efficient and appropriate health service use, and should aim to harness the potential benefits of the Internet, informed by an understanding of how and why people go online for health.

## Introduction

In the United Kingdom, in 2009, 70% of households had Internet access [1]. With this rise has come an increase in health-related Internet use. The proportion of UK Internet users using online information on health matters increased from 37% in 2005 to 68% in 2009 [1,2]. A survey in seven European countries in 2005 found that 71% of all adults reported using the Internet for health information [3], and in the United States this figure is 61% [4]. Furthermore, public perceptions of the importance of the Internet as a source of health information have risen dramatically [5].

The National Health Service (NHS) Direct website is the main health advice and information website for patients and the public in the United Kingdom. It was launched in December 1999, and in 2009, there were 18 million visits to the website, compared with 1.5 million visits in 2001 [6]. At the time of this research it provided health advice (through symptom checkers, a health encyclopedia, and an online enquiry service), information on local health services, articles regarding healthy living and fitness, and many other features, including a pregnancy planner, support for long-term conditions, and access to information about health care abroad. Since mid-2009 it has worked with the NHS Choices service, and some of the features described above have migrated to other parts of the NHS Choices platform. 

There is limited information describing how and why people use online health information, or the effect of this on health status, although this literature base is growing [6-11]. In this study we used a mixed-methods approach to investigate the characteristics and motivations of online health information seekers in England. In theory, the Internet offers certain advantages as a health information resource. In particular, it provides convenient and anonymous access at any time, from any location, to a wide range of expert sources; and through virtual communities it can provide peer support and social interaction [12]. Health-related use of the Internet has been hailed as a tool to support the emergence of the informed and empowered health consumer, and a shift in the balance of power between patient and professional [13]. At the same time, concerns have been raised about the quality of information, the potential for unhelpful peer-to-peer interactions, and the exclusion of individuals who experience barriers to access [14,15]. In Table 1 we have summarized the main characteristics and potential public health benefits and challenges that have been proposed for health-related Internet use [12,16,17]. In this paper, by exploring the expressed reasons for seeking online health information, we hope to assess the extent to which the theoretical benefits and challenges of the health Internet are being realized in practice.

**Table 1:** Theoretical characteristics and potential public health benefits and challenges of health-related Internet use

Characteristics of the health Internet	Potential public health benefits	Potential challenges to public health
Vast quantity of information Unregulated Always on Accessible from anywhere Interactive Information can be captured, archived, and retrieved Content from both expert sources and user-generated sources Content can be free or paid for Users can organize in virtual communities	Public education Public empowerment supporting informed consumers engaged in their own care Connect people with others who have similar problems Online social support Reduce barriers (time, location, and cost) to accessing information and services Avoid the stigma of real-world consultation for certain problems Deliver interactive interventions, as well as information Integrated health services such as shared electronic records Reduced travel and carbon emissions	Misinformation leading to harm Misuse of accurate information or services such as e-pharmacy Exacerbation of inequalities in health caused by the digital divide Challenges to the authority of health professionals Disruptive behavior in virtual communities Social isolation of users Internet addiction of users Ergonomic effects of computer use and reduced physical activity

## Methods

### Design

A combination of questionnaire survey and semistructured interviews was used. A self-administered, open, cross-sectional survey of visitors to the NHS Direct website was undertaken using a link placed on the home page of the website. It was therefore a web-based opt-in survey of a convenience sample. Cookies prevented multiple submissions from one computer. Consent was given by participants entering an email address to be sent the link to the survey. There were no incentives to participation. The questionnaire had two aims: first, to identify the characteristics and motivations of users of the website; and second, to recruit potential participants for a qualitative interview study. The questionnaire included 15 questions (one question per screen) covering demographic and health status characteristics, reasons for using the website, and questions related to information-seeking behavior. There was no adaptive questioning or manipulation of item order. In the final part of the survey, the respondents were asked for consent to be contacted at a later date for an interview. The questionnaire was developed based on previous work. The instrument is included as an online appendix.

Interview participants were selected by maximum variation sampling with respect to demographic and health status characteristics of gender, age, ethnicity, those seeking help for acute and chronic illnesses, and those seeking help for themselves or for others. To minimize recall bias, while allowing sufficient time for participants to act on the information they had found, interviews were conducted within 1 to 2 weeks of use of the site. These semistructured interviews were undertaken via telephone, email, or instant messaging. Interviews were conducted by two interviewers, who were members of the research team (JP and NI). Anonymized interview transcripts were used for analysis. We used open-ended questioning and determined the order of questioning by the direction taken by each interview participant. Each interview usually began with a brief description of the interviewee’s last visit to the NHS Direct website and went on to explore their online health-related information needs and their information-seeking behavior under the following headings: motivation for using the NHS Direct website, the NHS Direct website itself, facilitators and barriers to online health seeking, role of the Internet compared with other sources of information, and consequences of using online health information. Demographic characteristics of each participant were also recorded (age, gender, ethnicity, educational attainment, and current or most recent occupation). The interview topic guide is included as an online appendix. Survey and interview data were held in password-protected files on password-protected computers. NHS ethics committee approval was obtained for both elements of this study. 

### Analysis

Questionnaire results were analyzed to provide summary descriptive statistics and cross-tabulations for which chi-square statistics were calculated to examine differences in proportions by demographic characteristics. No statistical corrections such as weighting were used, but nonresponders to individual questions were excluded from the analysis of those questions. All interview transcripts were read by three investigators (JP, NL, and JR), who familiarized themselves with the data through reading and reflection, and each independently undertook open coding of all transcripts [18]. Constant comparison was used to refine emerging conceptual categories, including a search for deviant cases. The investigators met to agree on a series of thematic codes that described a number of categories and subcategories. These agreed-on codes were reapplied to the transcripts, using NVivo software (version 8, QSR International Pty Ltd, Southport, UK).

## Results

### Questionnaire Survey

A total of 792 respondents completed at least part of the survey accessed via the homepage link. Results are presented as a proportion of the total number of responses to each question (the denominator therefore varies according to the individual question response rate). As can be seen from Table 2, 71.2% (534/750) of respondents were aged under 45 years, 67.4% (511/758) were female, and 37.7% (286/759) had a university degree or higher qualification. With respect to personal general health, 61.7% (474/768) rated it as good or very good (compared with a general population figure of 76%) [19]; 42.6% (322/755) reported having a long-standing illness, disability, or infirmity (very similar to the general population figure of 42%) [19]. 

Regarding reasons for seeking help, 69.8% (545/781) reported looking for information for their own health issue, while 22.0% (172/781) reported looking for information for someone else (8.2% were looking for both, 64/781). These proportions differed by gender, with women more likely than men to report seeking help for someone else (χ^2^_2_ = 6.35, *P* = .04; 33% of women, 168/509, versus 25.2% of men, 61/242, reported seeking information for someone else or for both themselves and someone else). There was a significant difference by age group for women, which was predominantly due to a higher number of women in the 56- to 65-year-old age group reporting looking for help for someone else compared with women in other age bands (χ^2^_7_ = 22.89, *P* = .002). There was no difference by age group for men (χ^2^_7_ = 10.65, *P* = .15). 

Of all respondents, 47.5% (366/770) reported seeking help for a new health issue, while 19.6% (151/770) reported seeking help for a long-standing issue; 17.1% (132/770) reported seeking help for both new and long-standing issues, and 15.7% (121/770) for neither. The commonest category of user was a person who reported seeking help for a new health issue, regarding their own health (257/770, 33.4%). A total of 44.9% (346/770) reported having already consulted a health professional (such as a general practitioner or nurse) about the problem for which they were using the NHS Direct website, and 6.1% (47/770) had previously consulted the NHS Direct telephone service about the issue they were currently looking up online. There were no significant differences in this previous consultation behavior by gender (χ^2^_1_ = 0.625, *P* = .43). Users in younger age groups (<36 years) were less likely to report having had prior consultation with a health professional before using the website (χ^2^_1_ = 24.22, *P* < .001); 35.9% (143/398) of those aged up to 35 reported having consulted prior to using the website, compared with 53.9% (186/345) of those over 35. 

**Table 2:** Survey responses by gender (missing data reported because partially completed surveys were included in the analysis). Total respondents N = 792

		Female	Male	Missing data
**Age group (years)**				
	Under 25	184	56	1
	26-35	109	47	3
	36-45	91	41	2
	46-55	59	38	3
	56-65	40	34	1
	66-75	17	10	0
	Over 75	2	12	0
	Missing data	9	9	24
**Highest qualifications**				
	None	57	36	4
	O levels or equivalent	119	46	3
	A levels or equivalent	108	33	2
	University degree or equivalent	181	100	5
	Other	36	27	2
	Missing data	10	5	18
**State of general health (self-reported)**				
	Very good or good	306	153	15
	Fair	150	64	1
	Bad or very bad	50	25	4
	Missing data	5	5	14
**Looking for information for**				
	Myself	341	181	23
	Someone else	120	49	3
	Myself and someone else	48	12	4
	Missing data	2	5	4
**Looking for information on**				
	New issue	243	109	14
	Long-standing issue	88	59	4
	Both a new and long-standing issue	93	39	0
	Other	80	34	7
	Missing data	7	6	9
**Had previously consulted a health professional about this same issue**				
	Yes	230	103	13
	No	274	139	11
	Missing data	7	5	10

### Interview Sample

Twenty-six (20 female, 6 male) participants aged 16 to 75 years were recruited from a total of 265 who had indicated their willingness to take part in an interview on the questionnaire. They were interviewed either by telephone (n = 23) or by instant messaging/email (n = 3). Twenty-one described their ethnicity as white British, and five belonged to other ethnic groups. At the time of recruitment the participants were looking up information for themselves (n = 15), for someone else (n = 10), or for both themselves and someone else (n = 1). The participants rated their health as very good (n = 2), good (n = 6), fair (n = 14), bad (n = 1), or very bad (n = 3). Participant characteristics are summarized in Table 3.

The range of health topics searched for was broad, with the most popular being musculoskeletal problems (6/26), mental health problems (3/26), and dermatological problems (3/26).

**Table 3:** Characteristics of interview participants (n = 26)

		Female (n = 20)	Male (n = 6)
**Age group (years)**			
	16-25	4	0
	26-35	2	0
	36-45	4	2
	46-55	4	1
	56-65	4	3
	Over 65	2	0
**State of general health (self-reported)**			
	Very good or good	7	1
	Fair	9	5
	Bad or very bad	4	0
**Looking for information for**			
	Self	9	6
	Other	10	0
	Self and other	0	1

### Thematic Analysis of Interview Data

Themes were identified under the following headings, framed by the questions used in the interview topic guide: the motivations for seeking help online; the benefits of seeking help in this way, and some of the challenges faced; the strategies employed in navigating online health information provision and determining what information to use and to trust; and finally, specific comments regarding the NHS Direct website service. These will be discussed in turn.

#### Motivations

Within the category of motivations, four concepts emerged through the thematic analysis: the desire for reassurance; the desire for a second opinion to challenge other information; the desire for greater understanding to supplement other information; and perceived external barriers to accessing information through traditional sources (including the desire to avoid “bothering” their health care provider).

One prominent reported motivation for seeking online health information was reassurance, often at the time symptoms appeared and prior to consultation with a health professional. As one participant stated *“sometimes you just want your fears eased”* [Interviewee 25, a 41-year-old woman]. In general, this online search for reassurance and relief from anxiety was not said to replace other forms of seeking help; rather, it was seen as an adjunct to other sources of help and information, not a substitute for them, thereby providing an extra layer of information but not necessarily altering consulting behaviors. As Interviewee 19 put it:

Interviewee 19, a 48-year-old man I think I probably followed a course of action I would have taken anyway.

For the most part, the interviewees in this study reported seeking health information from official health websites that gave authoritative health information, and not from other users. This was not surprising given the route of recruitment through their use of the NHS Direct website. Even so, among the participants in this sample a few participants did report seeking nonprofessional “peer-to-peer” information. In these cases the motivation was again reassurance – wanting to know that the person was not alone in what they were experiencing. This was illustrated by Interviewee 17:

Interviewee 17, a 49-year-old woman I’ve gone to a menopause site that specializes in that [peer-to-peer interaction]. There are lots and lots of contributors over several years and I search for that, have a read and see what other people think and then I’ve posted on that and said“Look, I don’t have what people classically call flushes”... I just have it at night. What do other people think? And then lots of people come on and say“Yes, that’s perfectly normal. That’s what I’ve experienced and this is not unusual. People do have...”...so quite often I get reassurance that I’m not an odd one out from this.

A few examples of the Internet providing “demand management” for primary care or emergency services were identified. Sometimes participants described the motivation of not wanting to “*bother*” their doctor with a problem that might be trivial. Interviewee 13 described how reassurance over a bloodshot eye eliminated the need for a general practice appointment.

Interviewee 13, a 66-year-old woman It looks as if you should do something about it. I looked it up again on the website and it said “there’s nothing to worry about,” you know...it normally goes off on its own and it doesn’t need a trip to the doctor’s so again, it saved the trip to the doctor and it saved a lot of worrying.

However, while 13 interviewees talked about accessing the NHS Direct website as one of their first actions to find out information about symptoms, in most cases they did report going on to seek help from traditional health service sources, albeit sometimes with less urgency or less anxiety. This was the case whether they reported seeking help for themselves or for someone else. Furthermore, half the interviewees also stated that they would tend to see a professional as a first point of call if they had a health problem

Interviewee 9, a 32-year-old woman As I say, it didn’t make me do anything that I wasn’t already contemplating, but I guess it gave me the answer to the question that I was looking for and then the peace of mind that it wasn’t becoming an emergency I guess and that we should stick with it.

Where demand management appeared to be occurring in practice, this was usually explained by the avoidance of barriers to accessing traditional health services, such as difficulties in getting an appointment or in travelling to one.

Interviewee 10, a 25-year-old woman It used to take two hours and two bus journeys to get to the doctors...It’s easier to use than to get down to the doctor’s...It’s the time and the money...you know – those kinds of factors and then all the problems with getting appointments as well.

The desire for a second opinion following initial advice received from a heath professional was another reported motivation. Participants described the Internet as a way of accessing specialist knowledge, which they could use to challenge the advice given during their consultation. As in the example below from Interviewee 21, this challenge was an explicit response to not believing the health professional. At other times, illustrated by a quote from Interviewee 5, this was more about becoming fully informed on the range of divergent professional opinion.

Interviewee 21, a 59-year-old man They were telling me that treatment would be such and such and I thought“well I don’t believe that and I’ll use NHS Direct to see whether they can give me some information.”

Interviewee 5, a 55-year-old woman Well, when you go to the doctor you’ve only got his opinion haven’t you? I mean I’m sure he’s basing it on knowledge and research and things like that, but I just wanted to see other opinions about it.

The next motivation described by participants was also related to researching information prior to or following a consultation, but was not motivated by a desire to challenge. Instead the reported motivation was to seek clarity and confirmatory information in greater depth. This could be characterized as “homework” to support informed decision making, which could be done at the individual’s own pace. As Interviewee 20 explained:

Interviewee 20, a 62-year-old man It [the Internet] is excellent for a slower time study of information that my doctor hasn’t fully explained.

Interviewee18, a 46-year-old woman I was really looking to substantiate a little bit more about the treatment options that I was given by the GP.

#### Benefits and Challenges: Convenience, Coverage, and Confidentiality

Interviewees volunteered a range of benefits of online health information seeking. These reported benefits clustered around three theme areas: convenience, coverage, and anonymity. The convenience of online health encompassed the ease and speed of access, at any time, and from any location, especially from home. Access could be *“in your own time* *...* *at your own pace”* [Interviewee 19, a 48-year-old man]. This was contrasted with issues in accessing traditional health services.

Interviewee 24, a 58-year-old woman You can go on it any time of the day quite honestly, whereas you can’t get your doctor any time of the day, or they have to ring you back and then you’re sitting either waiting or...you know.

Interviewee 25, a 41-year-old woman It’s quick and it’s direct. It’s there in front of me every day and every evening.

In their responses, interviewees recognized that the Internet played a role in allowing them to become informed consumers, better able to share decisions with their health care provider. For example, Interviewee 18 (a 46-year-old woman) reported being able to *“make an informed decision”* in conjunction with her specialist, about treatment for fibroids, having sought information online. The benefit of coverage related to the wide range and depth of health information available on the Internet, and access to specialist medical knowledge. This access to esoteric medical knowledge was highlighted by Interviewee 4 (a 59-year-old woman):

Interviewee 4, a 59-year-old woman It’s [the Internet] the perfect tool for finding out something that you need to know about and you probably don’t have the information unless you’re a medic.

Confidentiality was the third area that emerged from the interviews as a key valued benefit for Internet health users. This encapsulated both the anonymous nature of online identity and the ability to use the Internet privately from any location. This could be of particular value for conditions that were more personal or stigmatizing.

Interviewee 22, a 20-year-old woman Confidentiality...you’re not speaking to someone about health issues. I mean for someone that has a lot more of a personal problem and they didn’t really want to discuss it with someone it’s ideal.

Interviewees also discussed some of the challenges of health-related Internet use but no prominent theme emerged. The issues raised included (1) inaccurate information leading to harmful health decisions, which was reported as more of a theoretical problem, rather than by anyone with direct experience; (2) misuse of accurate information, leading to inappropriate self-diagnosis: *“there’s always the worry of misdiagnosing something, or reading something into it”* [Interviewee 19, a 48-year-old man]; (3) confusion caused by the sheer volume of information, which was sometimes perceived as being *“confusing”* and *“daunting”*; and (4) sometimes criticism of health-related Internet use for its *“impersonal”* nature, which lacked the quality of face-to-face contact, and which could not replace a real consultation.

#### Strategy: Navigating the Health Internet

Throughout the interviews, participants explained the strategies employed in navigating online health provision and determining what information to use and to trust. They reported finding NHS Direct Online either by using a search engine (almost always this was Google), or by going directly to the uniform resource locator (URL) once they were familiar with it. Choosing a site was regarded as a common sense activity by interviewees. They were well aware that the Internet could be a source of misleading information – *“there’s a lot of crap on the Internet* *,* *”* as Interviewee 21 (a 59-year-old man) put it – but they used common sense to avoid this. For Interviewee 1, the Internet provided her with:

Interviewee 1, a 59-year-old woman A vast range of information from the idiotic to the academic, so I’ve got a vast range of information and it’s on tap so to speak. It’s up to the individual to adjudicate whether the information is relevant, or whether it’s valid...I do have enough common sense to evaluate what I see...I keep repeating that you need to use your discretion when you read...I would say that it could be a dangerous thing, on the other hand but I think the majority of people do have common sense.

Several participants expressed negative views regarding peer-to-peer sources of health information, related to concerns about its trustworthiness. For example, Interviewee 4 (a 59-year-old woman) was concerned that the information may be written by *“wild women from Minnesota* *,* *”* and Interviewee 5 explained:

Interviewee 5, a 55-year-old woman How do you know if they’re trustworthy? No, I wouldn’t do that at all. I don’t particularly like these chat rooms anyway...Because I don’t know them. No, I don’t want to talk to people I don’t know about things really. I think it could be quite dangerous...Perhaps I’m being cautious but that’s how I think.

“Brand recognition” was reported as very important to the interviewees in navigating the health Internet. Interviewees reported choosing sites that had “real-world” branding, that is, an identity that they recognized from their offline experiences. The importance of the brand in establishing that a site was trustworthy was a very strong theme across the interviews. The NHS brand in particular was seen by respondents as giving the website the valued qualities of being impartial, reliable, and up-to-date. As one interviewee put it *“You tend to trust the NHS don’t you?”* [Interviewee 11, a 38-year-old woman]. Interviewees often contrasted this inherent trustworthiness of the NHS brand with their views on commercial health websites, particularly those produced by pharmaceutical companies:

Interviewee 3, a 44-year-old woman I thought the NHS one probably has no axe to grind...Whereas if it’s related to a drug company or somebody with herbal medicine and all of this sort of thing, I think they tend to be more biased, whereas the NHS is not trying to sell you something.

A further interesting finding regarding which sites were valued and used was the low esteem in which North American sites were sometimes held as reported by our British participants. This was partly due to a perception that US sites had commercial aims and were therefore seen as *“trying to sell something*” and partly the lack of local or cultural relevance for some of the information.

Interviewee 25, a 41-year-old woman The thing about the NHS online is that you know you’re looking at genuine stuff. The answers that you’re going to get are absolutely spot on and you can rely and trust them, whereas if I just Google something I may end up on an American site or something. I wouldn’t feel confident that the information I was looking at was absolutely right.

#### The NHS Direct Service

As would be expected given the route for recruitment, respondents made many comments about specific aspects of the NHS Direct website; for example, wanting further information on specific topics. Over and above these individual remarks, two broad issues concerning the NHS Internet service were present across the interviews and had generic relevance to health-related Internet provision. The first of these was the perception that the NHS Direct website was integrated with the real-world NHS service. As Interviewee 4 (a 59-year-old woman) put it *“I would hope that it ties in with the NHS generally, so it seemed to be the sensible place to go* *.* *”* Interviewees had an expectation that there would be some connection between their use of a virtual health service and the care they received from the physical counterpart. Furthermore, they felt that, because they were NHS patients, using the NHS website was the “right thing to do,” because of this perceived integration across online and traditional services.

Interviewee 1, a 59-year-old woman Since we are under the NHS system, it would be logical for me to go first of all to the NHS Direct to see what the NHS’s take was...As far as I’m concerned, if the NHS Direct website is offering this information then it should make it uniform all through the NHS...I specifically used the NHS website because we live in an NHS world and where better than to get it straight from the horse’s mouth?

The second broad issue of generic relevance related to feedback about the clarity and simplicity of design of the NHS Direct website, in terms of the language used and the architecture of the site.

Interviewee 22, a 20-year-old woman It’s got clear information and there’s enough there, but not like reels and reels that you get that you struggle to understand it and it’s very well broken down into sections as well I think, so like very specific for children and adults.

Together with the issue of the NHS brand, which was described above under navigation strategy, the clarity of the site and the perceived link with the real-world service were the principal reasons reported for valuing the NHS Direct site in particular as a source of online health information.

## Discussion

The survey findings with respect to the age, gender, and educational status of online health seekers add to the accumulated evidence of several studies over the last decade that have shown that being female, being younger, and having a higher level of educational attainment are all associated with more frequent health-related Internet use [11,19-23]. The reported health status profile of our survey participants appeared to be very close to that of the general population. Work in other countries has sometimes shown a tendency to overrepresent people with chronic illness among health Internet users [7,8,24,25], while others have not found this association [11,22]. The NHS Direct website is used for both acute and chronic problems, mild or serious, and is used by individuals for themselves and on behalf of family members, especially by women. It is therefore perhaps not surprising that the health status of the users in our study was similar to that of the general population. The majority of those surveyed were seeking information for themselves, which is consistent with the findings of others [11]. Furthermore, a large proportion of users (over 40%) had already sought help from a health professional for the same health issue prior to accessing the website.

Having established the characteristics of the users of the site, we undertook in-depth qualitative work to explore in detail their motivations and attitudes. In 2003, the lead author (JP) wrote a review paper that summarized the benefits and challenges of health-related Internet use [12]. In Table 1 we integrated these with the thoughts of other authors in this field [16,17]. The analysis of our interviews supports most of the theoretical benefits discussed in the literature, and indicates that the health Internet is delivering on its potential benefits, while at the same time presenting some challenges to health professionals. Participants’ responses indicated that the Internet was being used as a tool to educate and reassure, and to sometimes challenge information received by health professionals. Previous work on the sociology of health-related Internet use has invoked theories of empowerment, democratization, and the challenge to health professional power [13,15,26] Most empirical work has indicated that, while these processes are taking place, the change is more subtle than many theorists have predicted, with the ongoing predominance of a biomedical model in the context of more-informed health consumers [27-29]. By sampling users of the NHS website it is perhaps not surprising that our findings support this model of evolutionary rather than revolutionary change, with health e-consumers seeking to become more informed through authoritative advice from official websites. Health-related Internet use was seen by most of our participants as a supplement to existing health service provision rather than a replacement for it [4,30]. The motivations of reassurance and of seeking greater understanding can be seen in this context. Even the motivation to find a second opinion to challenge other information was within the context of a model of biomedical authority. Our findings support the idea that online health resources are enmeshed with other (offline) approaches to seeking help [26], and that health-related Internet use is now embedded in everyday health practices [31]. 

The majority of online events were related to real-world consultations, whether as preparation for them or as a search for further information afterward [23,32,33]. There were few examples of demand management occurring in practice, in terms of reducing the need for consultations, but our findings do support the idea that a health website can lead to more appropriate use of other services. Peer-to-peer interaction was not a focus of this study, as this is not provided on the NHS Direct website, and the number of participants in our qualitative sample reporting use of online support groups for health conditions was not high. Nevertheless, some participants did discuss the value of online interaction with others with similar problems, in particular the reassurance of knowing they were not alone, as found in previous work [34], while others expressed concerns about the trustworthiness of peer-to-peer sources. Consumer access to poor-quality information on the Internet has been a long-standing concern in the eHealth literature [15]. We found that, in avoiding misinformation and identifying which information to trust, participants put great emphasis on recognition of brands such as the NHS, which were trusted in the non-Internet world, together with using common sense approaches to navigate the health Internet. The reported value of official branding of health websites in determining trustworthiness is supported by previous work [35]. The online benefits of convenience and anonymity are well established [36] and were widely reported, as was the expectation that online health services would be fully integrated with their real-world counterparts, something that remains an aspiration for the NHS in the United Kingdom but is not yet a reality.

Some theoretical benefits and challenges were not prominent in these interviews. The “green” potential of the Internet to reduce travel, but at the same time possibly reduce physical activity and lead to social isolation or depression [37], was not discussed by our participants. Nor were any ergonomic effects of computer use, or the problem of Internet addiction. The issue of the digital divide, although mentioned by three participants, did not emerge as a consistent theme. However, given that this sample were all Internet users, this was perhaps not surprising.

### Limitations

Because this was an opt-in survey accessed via a weblink, it was not possible to calculate a response rate for the questionnaire. There were also design issues with the website during the survey period, which meant that the link to the survey was not always clearly visible to users. This affected the overall response and the number of individuals consenting to be interviewed in the second stage. To minimize social desirability bias, the researchers made it clear to interviewees that the researchers were independent of the NHS Direct organization, but those volunteering for interviews may still have been a particular population who wanted to relate their experiences with NHS Direct, good or bad. More women than men were interviewed due to having very few male volunteers. Interview methodology of this type, asking people to report how and why they used a particular source, may reflect attitudes rather than actual behavior, for which direct observation may be preferred. Nevertheless, questions were designed to focus on the most recent actual use of the Internet for health, rather than rely on hypothetical questioning. The participants were users of the NHS Direct website and were therefore not necessarily representative of the overall population of online health information seekers in the United Kingdom. However, the NHS site is the most popular health information site in the United Kingdom, and the demographic profile of respondents was similar to that of non-UK-based studies.

### Conclusions

Given increasing resource constraints, the health care community needs to seek ways of promoting efficient and appropriate health care use, which should include consideration of how Internet health information is provided and used, and how traditional NHS services and online services can be best integrated. The study findings support a model of evolutionary rather than revolutionary change in health information use, with real-world trusted brands being used online, in conjunction with traditional consultations. It will be interesting to see whether in time, particularly as the younger “Internet generation” ages and eHealth literacy increases in all age groups [38], Internet health information will be trusted enough to be used as an alternative, as opposed to an adjunct, to other types of health-seeking activities, and by individuals of broader demographic profiles. Our findings fit with a “shared decision-making” model [39], where individuals seek information to help the decision-making process and confirm what they are being told, rather than seeking to become independent experts. One of the primary motivations was the seeking of reassurance, and the value of this in terms of health or social benefit or more appropriate service use needs to be further explored. The relationship between Internet use and health outcomes is an area for research development, including examination of the role of user empowerment. Health service providers should aim to harness the potential benefits of health-related Internet use, rather than see it as a burden or challenge.

## Abbreviations

NHS: National Health Service
URL: uniform resource locator
